# Dating app use and depression symptoms in adolescents

**DOI:** 10.1186/s13104-026-07641-9

**Published:** 2026-01-21

**Authors:** Jason M. Nagata, Sydnie K. Domingue, Thang Diep, Christiane K. Helmer, Abubakr A. A. Al-Shoaibi, Kyle T. Ganson, Alexander Testa, Jinbo He, Fiona C. Baker, Jason M. Lavender

**Affiliations:** 1 Department of Pediatrics, University of California, 550 16th Street, 4th Floor, Box 0503, San Francisco, CA 94143 USA; 2 Factor-Inwentash Faculty of Social Work, University of Toronto, 246 Bloor St W, Toronto, ON M5S 1V4 Canada; 3 Department of Management, Policy and Community Health, University of Texas Health Science Center at Houston, 7000 Fannin St, Houston, TX 77030 USA; 4 Department of Biosciences and Bioinformatics, School of Science, Xi′an Jiaotong-Liverpool University, 111 Ren′ai Road, Suzhou Dushu Lake Science and Education Innovation District, Suzhou Industrial Park, Suzhou, 215123 Jiangsu P. R. China; 5 Center for Health Sciences, SRI International, Menlo Park, CA 94025 USA; 6 School of Physiology, University of the Witwatersrand, 1 Jan Smuts Avenue, Braamfontein 2000, Johannesburg, South Africa; 7 Military Cardiovascular Outcomes Research Program (MiCOR), Department of Medicine, Uniformed Services University of the Health Sciences, 4301 Jones Bridge Rd, Bethesda, MD 20814 USA; 8 The Metis Foundation, 84 NE Interstate 410 Loop # 325, San Antonio, TX 78216 USA

**Keywords:** Adolescent, Youth, Online dating, Screens, Media, Dating, Mental health, Depression

## Abstract

**Objectives:**

The popularity of dating apps has grown significantly among younger demographics. Despite evidence on the relationship between online dating and mental health in adults, little research exists on underage online dating app use in adolescents. This study examines the association between dating app use and depression symptoms in a U.S. sample of 13–16-year-old adolescents.

**Results:**

We examined cross-sectional data from Year 5 (2021–2023) of the Adolescent Brain Cognitive Development (ABCD) Study (*N* = 11,530). Multivariable linear regression analyses using cluster-robust (heteroskedasticity-consistent) standard errors were used to estimate the association between dating app use and depression symptoms, adjusting for potential confounders including sociodemographic factors (age, sex, race/ethnicity, household income, parent education) and study site. In this demographically diverse sample of adolescents (47.9% female, 47.3% racial and ethnic minority), 0.9% reported ever using a dating app. Dating app use was associated with higher depression symptoms (standardized beta coefficient [ß] 0.42; 95% confidence interval [CI] 0.08–0.76; *p* = 0.017) in adjusted models. This association may be due to victimization and unsafe online activities. Future prospective and mechanistic studies are needed to better understand the link between underage dating app use and depression symptoms.

**Supplementary Information:**

The online version contains supplementary material available at 10.1186/s13104-026-07641-9.

## Introduction

Online dating has become a mainstay for finding sexual and romantic partners through the use of websites and mobile applications [1], transforming how adults socialize, date, and form romantic relationships. Despite the minimum age requirement of 18 for most online dating apps [2], the use of online dating platforms and apps is common among younger demographics. One study of youth from the U.S. found that 19% of adolescents under the age of 18 reported using (currently or ever) dating sites or apps [3]. The use of any dating sites and apps among underage adolescents is concerning, given the potential risks for online victimization and psychological distress [8–12, 14]. For instance, research in young adults indicates that online dating platforms expose users to risks associated with privacy [4], sexual abuse [5], and sexually transmitted diseases [6]. Moreover, a study of U.S. college students found that a higher frequency of sexual violence and harassment encountered via dating apps was correlated with lower self-esteem and higher depression and anxiety symptoms [7]. Studies of adults also have found that those who used dating apps were more likely to have higher levels of depression, social anxiety, and distress [8, 9].

There has been a relative paucity of studies examining associations between underage dating app use and mental health among adolescents. One study of Finnish, American, Spanish, and South Korean adolescents ages 15–18 found that those who engaged in online dating were more likely to experience online sexual harassment and victimization by both adults and peers [10]. Another study in Taiwan found that 15% of middle school students who engaged in online dating and used online dating platforms reported higher levels of depression, anxiety, and stress [11].

The potential link between underage dating app use and depression is of particular concern for adolescents, who are vulnerable to depression symptoms [12]. These symptoms can increase the risk of functional impairment [13] and later substance abuse [14]. Depression in adolescents continues to become more common, with data from the National Surveys on Drug Use and Health showing that the percentage of youth aged 12–17 years who experienced one or more major depressive episodes in the past year increased from 8.1% in 2009 to 15.8% in 2019 [15] and 19.5% in 2022 [16]. Accordingly, the U.S. Preventive Services Task Force (USPSTF) recommends annual screening for depressive disorders in adolescents beginning at age 12 for early identification and treatment [17].

A recent investigation by our team using data from the U.S. Adolescent Brain Cognitive Development (ABCD) Study found that 0.4% of early adolescents (mostly 11–12 years of age) reported having ever used a dating app [18]. Given that adolescence is an important developmental period characterized by the emergence of sexual and romantic feelings [19] and vulnerability to the onset of mental health concerns such as depression symptoms [12], understanding the extent to which underage use of dating apps and poorer mental health in adolescents may be linked is important. As such, building on our previous study reporting on the prevalence of dating app use among early adolescents, the current investigation examined the relationship between the use of dating apps and depression symptoms in a demographically diverse, national sample of 13–16-year-olds.

## Methods

### Study sample

We examined cross-sectional data of adolescents aged 13–16 years from Year 5 (2021–2023) of the ABCD Study (6.0 release), a longitudinal study of brain development and health in adolescents from the U.S. In 2016–2018 (baseline; Year 0), 11,875 children were recruited from 21 demographically diverse sites across the nation. These participants were recruited mainly through elementary schools, selected via stratified probability sampling of U.S. schools within the 21 catchment areas. Institutional review board approval was granted by the University of California, San Diego, and at each study site. Written assent was provided by participants, and written informed consent was provided by caregivers.

Of those enrolled in the study, 3,305 were missing depression symptom data, 51 were missing dating app data, and 162 were missing sociodemographic data (Appendix A). The exclusion of these participants yielded a sample of 8,444 participants. Multiple imputation by chained equations was used to address missing data, using covariate information to impute dating app use and the depression symptoms data. Predictive mean matching was applied under a missing-at-random assumption, which we evaluated for the depression symptoms outcome prior to imputation. The final analytical sample was 11,530 participants. Appendix B compares the sociodemographics and prevalence of online dating in the sample before multiple imputation.

### Measures

Dating app use was evaluated as lifetime use based on adolescents’ response to the following question: “Have you ever used a dating app?” (yes, no, don’t know what that is). To evaluate depression symptoms, the DSM-Oriented Affective Problems Scale of the parent/caregiver-reported Child-Behavior Checklist (CBCL) was used [20]. Parents/caregivers responded to 13 items addressing their child’s behavior on a scale from 0 (not true) to 2 (very true/often true). The CBCL has shown robust validity and reliability [21, 22]. The current investigation utilized the CBCL DSM-5 Scale continuous sum scores for depression symptoms (scale range: 0–26) at Year 5 in our analysis.

### Statistical analyses

To evaluate model assumptions, we examined residual distributions and heteroskedasticity. As expected for the CBCL depression symptoms score, residuals showed right skew and a large proportion of zeros. To account for potential heteroskedasticity and non-normality, all models were estimated using heteroskedasticity-robust standard errors clustered at the family level. For the primary analysis, multivariable linear regression models with standardized beta coefficients were used to estimate the association between dating app use and depression symptoms, adjusting for the following covariates: age, sex (female or male), race and ethnicity (Asian, Black, Latino/Hispanic, Native American, White, or other), household income (grouped into six categories reflecting the US median household income), highest parental education (high school or less, college education or more), and the ABCD study site. Appendix C includes detailed descriptions of each variable. Sensitivity analyses with additional covariates (daily recreational screen time, cyberbullying victimization, pubertal status, parental screen monitoring, age at first phone owned, average sleep duration, and relationship status) were conducted (Appendix D). Parallel analyses with Year 5 adolescent-reported internalizing symptoms sum score as the outcome were run (Appendix E), as data were not available for adolescent-reported depression symptoms. Statistical significance was based on a two-sided *p* < 0.05. We conducted precision/stability checks as well as leave-one-site-out analyses, and the pattern of results remained stable across all specifications. Survey weights were not applied because the available baseline weights are not intended for use with Year 5 data. Consequently, findings represent the unweighted Year 5 sample and may differ from population-weighted estimates. This study followed the Strengthening the Reporting of Observational Studies in Epidemiology (STROBE) reporting guidelines.

## Results

Characteristics of the 11,530 ABCD Study participants (mean age 15.0 years [SD = 0.6], 47.9% female, and 47.3% racial and ethnic minorities) are presented in Table 1. Overall, 0.9% reported ever using a dating app. The mean depression symptoms sum score was 1.9 (SD = 2.9).

The adjusted association with online dating and depression symptoms scores among ABCD Study participants is presented in Table 2. Adolescents who reported ever use of a dating app were significantly associated with higher depression symptoms (standardized beta coefficient [ß] 0.42; 95% confidence interval CI 0.08–0.76; *p* = 0.017).

**Table 1:** Sociodemographic, online dating, and depression characteristics of Adolescent Brain Cognitive Development (ABCD) Study participants (*N* = 11,530)

Sociodemographic characteristics	Mean (SD), range / %
Age (years)	15.0 (0.6), 13.3–16.7
Sex	
Female	47.9%
Male	52.1%
Race and ethnicity	
Asian	6.1%
Black	19.9%
Latino/Hispanic	17.0%
Native American	3.5%
Other^a^	0.9%
White	52.7%
Household income	
$24,999 or less	12.7%
$25,000 to $49,999	12.6%
$50,000 to $74,999	11.2%
$75,000 to $99,999	11.4%
$100,000 to $199,999	33.3%
$200,000 and greater	18.8%
Parent’s highest education	
High school education or less	14.7%
College education or more	85.3%
Online dating app use	
Never used	98.8%
Used	0.9%
Don’t know what it is	0.3%
Depression symptoms sum score	1.9 (2.9), 0–23

Results reflect estimates after multiple imputation using covariate-based imputations for online dating app use and depression symptoms sum score

^a^This subcategory was labeled ‘Other,’ with no predefined racial or ethnic groups; write-ins were allowed. Participants identifying as multiracial (and not Hispanic) or as a race/ethnicity other than Asian, Black, Latino/Hispanic, Native American, or White were included in this category

In sensitivity analyses, when adjusting for additional covariates (daily recreational screen time, cyberbullying victimization, pubertal status, parental screen monitoring, age at first phone owned, average sleep duration, and relationship status), ever use of a dating app was significantly associated with higher depression symptoms (ß 0.44; 95% CI 0.03–0.85; *p* = 0.035) (Appendix D). Ever use of a dating app was significantly associated with higher self-reported internalizing symptoms (ß 0.66; 95% CI 0.34–0.99; *p* < 0.001) (Appendix E).

**Table 2:** Cross-sectional associations between online dating app use and depression symptoms in the Adolescent Brain Cognitive Development (ABCD) Study at Year 5 follow-up (*N* = 11,530)

	Depression symptoms sum score			Depression symptoms sum score		
Online dating app use	Unadjusted ß (95% CI)	SE	p	Adjusted ß (95% CI)	SE	p
Never used	reference			reference		
Used	**0.47 (0.12**,** 0.82)**	**0.18**	**0.009**	**0.42 (0.08**,** 0.76)**	**0.17**	**0.017**
Don’t know what that is	-0.02 (-0.39, 0.35)	0.19	0.921	0.04 (-0.33, 0.41)	0.19	0.817

Bold indicates *p* < 0.05. ß=standardized beta coefficient from linear regression. SE = Cluster-robust (heteroskedasticity-consistent) standard errors clustered at the family level. Models represent the abbreviated output from the linear regression using multiple imputation, adjusted for age, sex, race and ethnicity, household income, parent education, site

## Discussion

Despite the majority of dating apps requiring users to be 18 years of age [3], we found that even young adolescents endorse engaging with these apps [18]. In a demographically diverse group of 13–16-year-old early adolescents in the U.S., the current study found that underage dating app use was significantly associated with greater depression symptoms. This result is consistent with another cross-sectional study that also found a significant association between dating app use and depression symptoms in adults [8].

There are several potential explanations for a link between dating app use and depression symptoms in adolescents. For example, the use of such apps may be related to validation-seeking behavior and social comparison, which are already heightened during early adolescence and can exacerbate feelings of inadequacy and low mood [8, 23]. Moreover, adolescence is a critical developmental stage that is characterized by significant emotional, physical, social, and behavioral changes, thus increasing vulnerability for adverse impacts of online dating [24]. Consistent with lifestyle exposure theory, adolescents who adopt online “dating lifestyles” may be more vulnerable to cyberbullying, privacy breaches, harassment, and other forms of online victimization [10], all of which have been linked to increased depression, anxiety, and psychological distress [11].

Importantly, the current findings were cross-sectional, so directionality or causality could not be established. It is also possible that there are bidirectional linkages in which adolescents with greater depression symptoms are more likely to use online dating apps, perhaps as a way to address loneliness or isolation. Depression symptoms often reduce energy levels and minimize the urge to engage socially, resulting in social avoidance and fewer opportunities to initiate relationships [25]. Building relationships online may be more comfortable than face-to-face relationships due to lower social pressures, online anonymity, and the ability to edit messages and control self-presentation, creating a positive experience for adolescents who would otherwise struggle to connect with peers [25]. Additionally, adolescents experiencing depression are more likely to engage in rule-breaking [26] and risky behaviors [27, 28], which may include underage use of online dating apps.

Finally, it is also possible that other factors like personality traits that were not explored in the current study may underline both vulnerability to depression symptoms and propensity for engaging in prohibited behaviors (such as underage dating app use). For example, neuroticism generally peaks in adolescence and then declines with age [29]. This personality trait is consistently linked to internalizing psychopathology, negative affectivity, and heightened sensitivity to rejection [30]. Given that adolescents are still developing regulatory and coping systems, they may be even more sensitive to digital social evaluation and rejection. In contrast, neuroticism has also been connected to impulsivity [31], a key predictor of risky behaviors in adolescents [32]. As such, adolescents who are higher in neuroticism may be more likely to engage in exploration of age-inappropriate digital spaces. These personality traits may therefore shape both mental health and patterns of digital engagement in early adolescents.

Our study had several strengths. It was the first to find an association between underage dating app use and depression symptoms in adolescents. Additionally, our overall sample was large and demographically diverse.

This study also had several limitations. First, depression symptoms were measured using a parent-reported single item, which may underestimate or misclassify internalizing symptoms. It has been observed that there are often discrepancies between children’s self-reports and their parents’ reports on mental health indicators, particularly for internalizing symptoms [33]. However, we included adolescent-reported internalizing symptoms sum score as an outcome for a parallel analysis, and results were similar. Second, the assessment of dating app use in this analysis was based on a single survey question, which did not evaluate for recency, frequency, motivations, or platform types. There was also no information on whether accounts were verified or age-gated, though the minimum age requirement is 18 for most online dating apps [2]. Although ABCD collected data on whether participants were currently using a dating app (4 participants, 0.0%) and how much time per week they spent on dating apps (0.4% reported > 0 min), prevalence was extremely low. This is not unexpected given the participants’ age and the likelihood of underage use occurring outside formal reporting. These exposure measures would likely have demonstrated stronger associations with depression symptoms due to greater temporal overlap. Third, although we controlled for demographic and contextual factors, residual confounding from unmeasured factors may contribute to the observed association between underage dating app use and depression symptoms. However, results stayed similar in the sensitivity analyses additionally adjusted for daily recreational screen time, cyberbullying victimization, pubertal status, parental screen monitoring, age at first phone owned, average sleep duration, and relationship status. Finally, the number of participants who endorsed dating app use was small, thus resulting in potentially limited power for the adjusted analysis.

Future research should address several methodological priorities to clarify the relationship between adolescent dating app use and mental health. Studies should utilize adolescent self-reports to obtain a more accurate assessment of internal experiences and depression symptoms, which may be underrecognized by caregivers. To move beyond single-item measures, investigators should use multidimensional instruments that capture key aspects of dating app engagement—including primary motivations and exposure to negative interpersonal experiences such as cyberbullying or harassment. Longitudinal designs are also essential for determining the temporal sequence of these relationships, including whether depression symptoms precede dating app use, whether app use contributes to later psychological distress, or whether these variables reciprocally impact one another over time. Finally, enhancing statistical power and improving generalizability will require sampling strategies that focus on older adolescents or subgroups with higher rates of dating app use, while also incorporating assessment of recency and frequency of use among these adolescents.

## Conclusion

The results of the present study suggest the potential importance of assessing for and addressing underage dating app use in adolescents. The apparent link between online dating and depression symptoms suggests the utility of refining parental guidelines on digital media use, particularly with regard to supporting strategies that prevent and reduce exposure to unsafe online behaviors [34]. Strategies such as parental involvement and monitoring in adolescents have been reported to decrease online victimization and online risks [35], which in turn may decrease psychological distress and depression symptoms [11]. Parents, clinicians, and educators can also provide education on the risks and benefits of online dating and underage dating app use. Future research can investigate the directionality between underage dating app use and depression symptoms and aim to better understand motivations for dating app usage in adolescents.

## Supplementary Information

Supplementary Material 1.

## Data Availability

Data used in the preparation of this article were obtained from the ABCD Study (https://abcdstudy.org), held in the NIH Brain Development Cohorts (NBDC) Portal.

## Abbreviations

ABCD Study: Adolescent Brain Cognitive Development Study
