# Supplementary material for: Dating app use and depression symptoms in adolescents
Source: BMC Res Notes. 2026 Jan 21;19:69. doi: 10.1186/s13104-026-07641-9 (PMC12908280; doi:10.1186/s13104-026-07641-9)
Supplement: Supplementary file 1 — Supplementary Material 1. [file 13104_2026_7641_MOESM1_ESM.docx]

Appendix A. Flow chart of study selection based on the inclusion and exclusion criteria

11,875 participants in from baseline (Year 0, 2016-2018) of the Adolescent Brain Cognitive Development (ABCD) Study

3,305 excluded for missing depression symptoms data at Year 5

8,657 participants included with depression symptoms data at Year 5

51 excluded for also missing dating app data at Year 5

8,606 participants included with depression symptoms and dating app data at Year 5

162 excluded for also sociodemographic data at Year 5

8,444 participants included with complete depression symptoms, dating app, and sociodemographic data at Year 5

Note: This chart depicts the inclusion criteria and sample size prior to multiple imputation.

| Appendix B. Comparison of the sociodemographic characteristics of the Adolescent Brain Cognitive Development (ABCD) study participants included vs. excluded in the sample before multiple imputation | | | |
| --- | --- | --- | --- |
| Sociodemographic characteristics | Included (n=8,444) | Excluded (n=3,518) | p |
| Age (years) | 15.1 (0.7) | 15.1 (0.7) | 0.218 |
| Sex (%) |  |  | 0.844 |
| Female | 47.8% | 48.0% |  |
| Male | 52.2% | 52.0% |  |
| Race and ethnicity (%) |  |  | <0.001 |
| Asian | 6.1% | 5.8% |  |
| Black | 18.7% | 24.0% |  |
| Latino / Hispanic | 15.9% | 20.3% |  |
| Native American | 3.4% | 3.7% |  |
| Other | 0.9% | 1.0% |  |
| White | 55.0% | 45.1% |  |
| Household income (%) |  |  | <0.001 |
| $24,999 or less | 10.1% | 20.1% |  |
| $25,000 to $49,999 | 11.6% | 15.5% |  |
| $50,000 to $74,999 | 10.7% | 12.3% |  |
| $75,000 to $99,999 | 11.8% | 10.2% |  |
| $100,000 to $199,999 | 35.5% | 27.3% |  |
| $200,000 or greater | 20.3% | 14.6% |  |
| Parent's highest education (%) |  |  | <0.001 |
| High school education or less | 13.1% | 20.7% |  |
| College education or more | 86.9% | 79.3% |  |
| Online dating app use (%) |  |  | 0.744 |
| Never used | 98.9% | 99.2% |  |
| Used | 0.9% | 0.5% |  |
| Don't know what that is | 0.2% | 0.3% |  |

| Appendix C. Variable names and definitions used in the Adolescent Brain Cognitive Development (ABCD) Study and the coding scheme applied in the analysis | | | | |
| --- | --- | --- | --- | --- |
| Variable | ABCD variable name | Table name and variable description | Response categories | Operationalized coding |
| Online dating app use | nt_y_stq__dapp_001 | Screen Time Questionnaire [Youth Report]: Have you ever used a dating app? | 1 = Yes 0 = No 999 = I don't know what that is 777 = Decline to answer | 1 = Yes 0 = No 999 = I don't know what that is |
| Depression symptoms sum score | mh_p_cbcl__dsm__dep_sum | Child Behavior Checklist [Parent Report] (DSM-5 Oriented Scale - Depressive problems): Sum [Validation: No more than 0 missing or declined] | Continuous | Continuous |
| Age | ab_p_demo_age | Demographics [Parent Report]: Youth's age at data collection (note: if table-specific date/time is missing, the age at visit start is used) | Continuous | Last observation carried forward applied Continuous |
| Sex | ab_g_stc__cohort_sex | Cohort description [Parent Report]: Participant's sex | 1 = Male 2 = Female | 1 = Male 2 = Female |
| Race and ethnicity | ab_p_demo__ethn_001 | Demographics [Parent Report]: Do you consider the child Hispanic/Latino/Latina? | 1 = Yes 0 = No 777 = Decline to answer 999 = Don't know | Followed Add Health 6-category grouping 1 = Asian 2 = Black 3 = Latino/Hispanic 4 = Native American 5 = Other  6 = White |
|  | ab_p_demo__race_* | Demographics [Parent Report]: What race do you consider the child to be? [Multi-select]: | 18 multi-select options |  |
| Household income | ab_g_dyn__cohort_income__hhold__6lvl | Cohort description [Parent]: Household income - 6 levels | 1 = < 25k 2 = 25k to 50k 3 = 50k to 75k 4 = 75k to 100k 5 = 100k to 200k 6 = > 200k 777 = Decline to answer 999 = Don't know | Last observation carried forward applied 1 = < 25k 2 = 25k to 50k 3 = 50k to 75k 4 = 75k to 100k 5 = 100k to 200k 6 = > 200k |
| Highest parental education | ab_p_demo__edu__slf_001__v02 | Demographics [Parent Report]: What is the highest grade or level of school you have completed or the highest degree you have received? | 0 = Never attended/Kindergarten only 1 = 1st grade 2 = 2nd grade 3 = 3rd grade 4 = 4th grade 5 = 5th grade 6 = 6th grade 7 = 7th grade 8 = 8th grade 9 = 9th grade 10 = 10th grade 11 = 11th grade 12 = 12th grade, no diploma 13 = High school graduate 14 = GED or equivalent 15 = Some college, no degree 16 = Associate degree: Occupational, Technical, or Vocational 17 = Associate degree: Academic Program 18 = Bachelor's degree (ex. BA, AB, BS, BBS) 19 = Master's degree (ex. MA, MS, MEng, MEd, MBA) 20 = Professional School degree (ex. MD, DDS, DVN, JD) 21 = Doctoral degree (ex. PhD, EdD) 777 = Decline to answer 999 = Don't Know | Highest parent education out of the two parents selected and last observation carried forward applied 0 = High school or less 1 = College or more |
|  | ab_p_demo__edu__prtnr_001 | Demographics [Parent Report]: What is the highest grade or level of school your partner completed or highest degree they received? |  |  |
| Family ID | ab_g_stc__design_id__fam | Design/nesting: Family ID - Participants belonging to the same family share a family ID (derived based on caregiver information) | Unique ID per family | Unique ID per family |
| Study site | ab_g_dyn__design_site | Design/nesting: Assessment site | 1 = Children’s Hospital Los Angeles 2 = University of Colorado Boulder 3 = Florida International University 4 = Laureate Institute for Brain Research 5 = Medical University of South Carolina 6 = Oregon Health & Science University 7 = University of Rochester 8 = SRI International 9 = University of California, Los Angeles 10 = University of California, San Diego 11 = University of Florida 12 = University of Maryland, Baltimore 13 = University of Michigan 14 = University of Minnesota 15 = University of Pittsburgh Medical Center 16 = University of Utah 17 = University of Vermont 18 = University of Wisconsin, Milwaukee 19 = Virginia Commonwealth University 20 = Washington University in St. Louis 21 = Yale University 22 = Icahn School of Medicine at Mount Sinai | Last observation carried forward applied 1 = Children’s Hospital Los Angeles 2 = University of Colorado Boulder 3 = Florida International University 4 = Laureate Institute for Brain Research 5 = Medical University of South Carolina 6 = Oregon Health & Science University 7 = University of Rochester 8 = SRI International 9 = University of California, Los Angeles 10 = University of California, San Diego 11 = University of Florida 12 = University of Maryland, Baltimore 13 = University of Michigan 14 = University of Minnesota 15 = University of Pittsburgh Medical Center 16 = University of Utah 17 = University of Vermont 18 = University of Wisconsin, Milwaukee 19 = Virginia Commonwealth University 20 = Washington University in St. Louis 21 = Yale University 22 = Icahn School of Medicine at Mount Sinai |
| Internalizing symptoms | mh_y_bpm__int_sum | Brief Problem Monitor [Youth Report] (Internalizing): Sum [Validation: No more than 0 missing or declined] | Continuous | Continuous |
| Average recreational screen time  (only weekend day available at Year 5) | nt_y_stq__screen__wknd__tot__hr_001 | Screen Time Questionnaire [Youth Report]: On a typical weekend day (or non-school day, like during summer or holiday breaks), how much time per day do you spend in total on a computer, phone, tablet, iPod, or other digital/mobile technology or video game? Please do not include time spent on school related work, but do include watching TV, shows or videos, texting or chatting, playing games, or visiting social networking sites (e.g. Facebook, Twitter, Instagram): Hours | Continuous | Continuous, winsorzied at 16 hours |
| Cyberbullying victimization | mh_y_cb_001a__01 | Cyberbullying [Youth Report]: Have you ever been cyberbullied: Has this happened to you in the past 12 months? | 1 = Yes 0 = No 999 = I don't know what that is 777 = Decline to answer | 1 = Yes 0 = No |
| Pubertal status | ph_y_pds__f_categ | Pubertal Development Scale & Menstrual Cycle Survey History [Youth Report] (Female): Approximate tanner stages [Validation: No more than 0 missing or declined] | 1 = Pre pubertal 2 = Early pubertal 3 = Mid pubertal 4 = Late pubertal 5 = Post pubertal | 1 = Pre pubertal 2 = Early pubertal 3 = Mid pubertal 4 = Late pubertal 5 = Post pubertal |
|  | ph_y_pds__m_categ | Pubertal Development Scale & Menstrual Cycle Survey History [Youth Report] (Male): Approximate tanner stages [Validation: No more than 0 missing or declined] |  |  |
| Parental screen time monitoring | nt_p_stq_010 | Parent Screen Time Questionnaire [Parent Report]: I keep track of my child's screen time during the week | 1 = Strongly Disagree 2 = Somewhat Disagree 3 = Somewhat Agree 4 = Strongly Agree 777 = Decline to Answer | Continuous mean score of nt_p_stq_010 and nt_p_stq_011 |
|  | nt_p_stq_011 | Parent Screen Time Questionnaire [Parent Report]: I keep track of my child's screen time during the weekend |  |  |
| Age at first phone ownership | nt_p_yst_001__03 | Screen Time Questionnaire [Parent Report]: How old was your child when he/she got their own cell phone? | Continuous | Continuous |
| Sleep duration | ph_y_mctq__sleep_dur | Munich Chronotype Questionnaire [Youth Report] (Sleep): Duration (Weekly summary score) | Continuous | Continuous |
| Relationship status | ph_y_sex__rore_003 | Sexual Behavior, Orientation, & Communication [Youth Report]: Are you currently in a romantic relationship? | 1 = Yes 0 = No 777 = Decline to answer | 1 = Yes 0 = No 777 = Decline to answer |

| Appendix D. Cross-sectional associations between online dating app use and depression symptoms in the Adolescent Brain Cognitive Development (ABCD) Study at Year 5 follow-up (N=6,850) | | | | | | | | | |
| --- | --- | --- | --- | --- | --- | --- | --- | --- | --- |
|  | Depression symptoms sum score | | | Depression symptoms sum score | | | Depression symptoms sum score | | |
| Online dating app use | Unadjusted ß (95% CI) | SE | p | Adjusted^a^ ß (95% CI) | SE | p | Adjusted^b^ ß (95% CI) | SE | p |
| Never used | reference |  |  | reference |  |  | reference |  |  |
| Used | **0.61 (0.18, 1.03)** | **0.22** | **0.006** | **0.55 (0.14, 0.97)** | **0.21** | **0.009** | **0.44 (0.03, 0.85)** | **0.21** | **0.035** |
| Don't know what that is | -0.19 (-0.59, 0.22) | 0.21 | 0.375 | -0.01 (-0.44, 0.42) | 0.22 | 0.958 | -0.01 (-0.44, 0.42) | 0.22 | 0.960 |
| Bold indicates p<0.05. ß=standardized beta coefficient from linear regression. SE=Cluster-robust (heteroskedasticity-consistent) standard errors clustered at the family level. Models represent the abbreviated output from the linear regression using listwise deletion (only participants with data for all variables were included in this analysis). | | | | | | | | | |
| ^a^ Adjusted for age, sex, race and ethnicity, household income, parent education, site | | | | | | | | | |
| ^b^ Adjusted for age, sex, race and ethnicity, household income, parent education, site, average daily recreational screen time, cyberbullying victimization, pubertal status, parental screen monitoring, age at first phone owned, average sleep duration, and relationship status. | | | | | | | | | |

| Appendix E. Cross-sectional associations between adolescent-reported online dating app use and internalizing symptoms in the Adolescent Brain Cognitive Development (ABCD) Study at Year 5 follow-up (N=6,748) | | | | | | | | | |
| --- | --- | --- | --- | --- | --- | --- | --- | --- | --- |
|  | Internalizing symptoms sum score | | | Internalizing symptoms sum score | | | Internalizing symptoms sum score | | |
| Online dating app use | Unadjusted ß (95% CI) | SE | p | Adjusted^a^ ß  (95% CI) | SE | p | Adjusted^b^ ß  (95% CI) | SE | p |
| Never used | reference |  |  | reference |  |  | reference |  |  |
| Used | **0.71 (0.39, 1.03)** | **0.16** | **<0.001** | **0.66 (0.34, 0.99)** | **0.16** | **<0.001** | **0.52 (0.21, 0.82)** | **0.16** | **0.001** |
| Don't know what that is | -0.34 (-0.87, 0.19) | 0.27 | 0.214 | -0.01 (-0.43, 0.41) | 0.21 | 0.956 | -0.04 (-0.44, 0.37) | 0.21 | 0.865 |
| Bold indicates p<0.05. ß=standardized beta coefficient from linear regression. SE=Cluster-robust (heteroskedasticity-consistent) standard errors clustered at the family level. Models represent the abbreviated output from the linear regression using listwise deletion (only participants with data for all variables were included in this analysis). | | | | | | | | | |
| ^a^ Adjusted for age, sex, race and ethnicity, household income, parent education, site | | | | | | | | | |
| ^b^ Adjusted for age, sex, race and ethnicity, household income, parent education, site, average daily recreational screen time, cyberbullying victimization, pubertal status, parental screen monitoring, age at first phone owned, average sleep duration, and relationship status. | | | | | | | | | |
